# Single and Repeated Dose 28-Day and 13-Week Toxicity Studies of Oil Prepared from the Internal Organs of the Japanese Giant Scallop (*Patinopecten yessoensis*) in Mice

**DOI:** 10.3390/foods9060691

**Published:** 2020-05-27

**Authors:** Koki Sugimoto, Toshifumi Tanizaki, Eito Shimizu, Ryota Hosomi, Kenji Fukunaga, Munehiro Yoshida, Takeya Yoshioka, Koretaro Takahashi

**Affiliations:** 1 Faculty of Chemistry, Materials, and Bioengineering, Kansai University, 3-3-35, Yamate-cho, Suita, Osaka 564-8680, Japan; k561198@kansai-u.ac.jp (K.S.); k955640@kansai-u.ac.jp (T.T.); k780736@kansai-u.ac.jp (E.S.); fukunagk@kansai-u.ac.jp (K.F.); hanmyou4@kansai-u.ac.jp (M.Y.); 2 Research and Development Division, Food Technology Group, Hokkaido Industrial Technology Center. 379, Kikyo-cho, Hakodate, Hokkaido 041-0801, Japan; yoshioka@techakodate.or.jp; 3 Faculty of Engineering, Kitami Institute of Technology, 165, Koen-cho, Kitami, Hokkaido 090-8507, Japan; kore@mail.kitami-it.ac.jp

**Keywords:** omega-3 polyunsaturated fatty acid, eicosapentaenoic acid, docosahexaenoic acid, Japanese giant scallop (*Patinopecten yessoensis*), safety assessment

## Abstract

Internal organs of discarded scallops are rich in omega-3 polyunsaturated fatty acids, but it is not used as a food ingredient due to the presence of toxic substances. Recently, our research team prepared high-quality scallop oil (SCO) from the internal organs of the Japanese giant scallop (*Patinopecten yessoensis*), in which cadmium and diarrhetic shellfish toxin are below regulated levels. In this study, SCO was prepared from the internal organs of scallops obtained from Mutsu and Uchiura bays in Japan, and was referred to as SCO-M (scallop oil from Mutsu bay) and SCO-U (scallop oil from Uchiura bay), respectively. Acute and subacute toxicity studies were performed to assess the safety of the prepared SCO. In acute toxicity study, mice were orally administered SCO-M and SCO-U at a single dose of 5,000 mg/kg body weight. In a 28-day repeated oral dose toxicity study, the mice were fed diets containing 1% and 5% SCO-M and SCO-U; and in a 13-week repeated oral dose toxicity study, the mice were fed 5% SCO-M and SCO-U. There were no toxicologically significant changes in clinical signs, hematology, blood chemistry, and organ weights at any dose during the experiment. Therefore, it was concluded that SCO-M and SCO-U are safe for use as food ingredients under the experimental conditions of this study.

## 1. Introduction

Omega-3 polyunsaturated fatty acids (PUFA) including α-linolenic acid (ALA), eicosapentaenoic acid (EPA), and docosahexaenoic acid (DHA) play important roles in our body as components of phospholipid (PL), which forms cell membranes, and eicosanoids. Eicosanoids are locally acting signaling molecules that control inflammation, vasoconstriction, and platelet aggregation. Eicosanoids synthesized from omega-6 PUFA, such as arachidonic acid (AA), are generally more potent mediators than those synthesized from omega-3 PUFA, although there are a few exceptions [1]. Omega-3 and omega-6 fatty acids generally compete for the same enzymes; especially, EPA and DHA compete with AA for the synthesis of eicosanoids [2]. Intake of omega-3 PUFA improves the balance of eicosanoids and consequently helps reduce inflammation [3]. Moreover, omega-3 PUFA has been reported to reduce the risk of cardiac death in several epidemiological and clinical trials, partially due to its anti-inflammatory effects [4,5,6]. Therefore, various recommendations for the daily intake of omega-3 PUFA have been proposed.

Among vegetable oils, flaxseed oil is rich in ALA (49–60%) and contains a modest level of linoleic acid (LA, 12–17%) [7]. Flaxseed oil is thus one of the richest omega-3 PUFA sources among edible fats and oils. The bioconversion rate of ALA to EPA or DHA in humans is generally poor [8], reaching only 8% and <0.1%, respectively [9]. Several factors are thought to influence the bioconversion to EPA and DHA, especially the competition of LA with ALA for Δ6-desaturase, which is a rate-limiting enzyme in the bioconversion of LA to AA, and ALA to EPA and DHA [10]. Therefore, to reduce the risk of coronary heart disease, intake of EPA and DHA instead of ALA is recommended by the Food and Drug Administration (FDA) and the American Heart Association (AHA) [11,12].

EPA and DHA are present in several dietary supplement formulations, including fish oil, krill oil, and algal oil. A typical fish oil supplement consists of about 1000 mg/person/day fish oil, inclusive of 180 mg EPA and 120 mg DHA [13]. Reports on health-promoting functions of EPA and DHA have led to an increase in the demand for oils containing EPA and DHA. As fish is a restricted resource, there is growing interest in exploring alternative sources of EPA and DHA. From the perspective of fish resource protection, krill oil and algal oil are attractive alternatives. The biomass obtained from krill (*Euphausia superba*) is generally between 125 and 750 million metric tons [14], thus being attractive for commercial harvest. Although krill oil contains both EPA and DHA, their concentration is less than that of anchovy fish oil [15]. Algal oil, on the other hand, has advantages, such as acceptability to vegetarians and improved palatability and smell, but is relatively expensive [16]. Previous studies have demonstrated that the hepatopancreas of Japanese giant scallop (*Patinopecten yessoensis*) has extremely high omega-3 PUFA content, especially EPA, as compared to typical fish oils [17,18]. In Japan, quantities of around 500,000 tons of Japanese giant scallops are landed annually, but there is only about 15% (*w/w*) of the adductor muscle, which is the edible part. The internal organs of scallop, such as hepatopancreas, gonads, mantles, and gills, are generally discarded during processing and account for 32,000 and 6000 tons/years in Hokkaido and Aomori, respectively. The global production of *P. yessoensis* was 2200 thousand tons in 2016, and their hepatopancreas were discarded [19]. Thus, the abundance of scallop internal organs makes them attractive alternative sources of omega-3 PUFA. However, in particular, the scallop hepatopancreas contains large amounts of toxic components, such as arsenic (As) compounds, cadmium (Cd), and sometimes diarrhetic shellfish toxin (DST) [20,21]. For this reason, the scallop internal organs have not been utilized as sources of EPA and DHA. Recently, we successfully prepared high-quality scallop oil (SCO) that satisfies the specifications for use as food from the scallop internal organs, by removing toxic components, including As compounds, Cd, and DST [22]. Omega-3 PUFA accounts for 40% of the fatty acid composition of SCO, which is much higher than that of fish oil and krill oil. In this study, we prepared SCO using the previously described method [22] from the scallop internal organs obtained from two different processing areas and referred to them as SCO-M (SCO from Mutsu bay, Aomori, Japan) and SCO-U (SCO from Uchiura bay, Hokkaido, Japan). Although SCO-M and SCO-U are novel alternative sources of EPA and DHA, the safeties of SCO-M and SCO-U are uncertain due to the presence of toxic substances in hepatopancreas. Our previous study demonstrated that SCO-M and SCO-U did not exhibit genotoxicity in in vitro (Ames test) and in vivo (micronucleus test) studies [22]. However, no acute and sub-acute toxicity studies have been carried out in animal models. Therefore, further safety assessments of SCO-M and SCO-U are required for their use as food ingredients or supplements. In this study, the safeties of SCO-M and SCO-U were assessed in ICR mice by single oral dose toxicity test and repeated oral dose toxicity studies for 28 days and 13 weeks. In the repeated oral dose toxicity studies, the effects of tuna oil, which is already available in the market, were also investigated.

## 2. Materials and Methods 

### 2.1. Materials

The internal organs of the Japanese giant scallops (*P. yessoensis*) from Mutsu and Uchiura bays were provided by Sato Chikoro Co. (Aomori, Japan) and Yakumo fishery cooperative (Hokkaido, Japan), respectively. The internal organs of Mutsu bay scallops consisted of only hepatopancreas that were collected between October 2016 and November 2016. In contrast, the internal organs of Uchiura bay scallops included hepatopancreas, gonads, gills, and mantles that were collected between August 2017 and September 2017. SCO-M and SCO-U were prepared according to the method described previously [22]. Soybean oil was obtained from Merck KGaA (Darmstadt, Germany). Tuna (*Thunnus orientalis*) oil was provided by Yashima Shoji Co., Ltd (Shizuoka, Japan). The ingredients of the experimental diet were obtained from Fujifilm Wako Pure Chemical Co. (Osaka, Japan) and Oriental Yeast Co., Ltd. (Tokyo, Japan). All other chemicals were of reagent grade and were purchased from Nacalai Tesque, Inc. (Kyoto, Japan) and Merck KGaA.

### 2.2. Lipid Composition of the Experimental Oils

After methylation of fatty acids with boron trifluoride-methanol, the fatty acid composition of soybean oil, SCO-M, SCO-U, and tuna oil were determined using a gas chromatography (GC) system (GC-2014; Shimadzu Co., Kyoto, Japan) equipped with Omegawax^®^ capillary column (Merck KGaA) as described previously [23]. The PL content of the experimental oils was determined using phosphorus analyses as described previously [24]. Cholesterol content was measured by GC (GC-2014) equipped with a DB-5 capillary column (Agilent Technologies Japan Ltd., Tokyo, Japan) with an internal standard of 5-α-cholestane [25]. The α-Tocopherol (α-Toc) content was measured using a high-performance liquid chromatography (HPLC) system of Model Prominence Series (Shimadzu Co.) equipped with a reversed phase column (Inertsil^®^ ODS column, 250 × 4.6 mm I.D., GL Sciences Inc., Tokyo, Japan) [26]. The peroxide value and acid value of the experimental oils were determined as described previously [27]. In addition, Cd, As mercury, dioxin, pesticide residues, polychlorobiphenyl, and DST in SCO-M and SCO-U were analyzed using the official analytical methods performed by a commercial service (Japan Food Research Laboratories, Tokyo, Japan).

### 2.3. Ethics and Animals

The animal experiments were conducted in accordance with the “Guide for the Care and Use of Experimental Animals” issued by the Prime Minister’s Office of Japan and the experimental protocol was reviewed and approved by the Animal Ethics Committee of Kansai University (approval No. 1611 and 1704).

Four-week-old male and female ICR mice were purchased from Japan SLC, Inc. (Hamamatsu, Japan), and an acclimatization period of 7 days was provided to the animals. During acclimatization and experimental periods, mice were kept in an air-conditioned room (temperature: 21–23 °C; humidity: 50–70%; illuminated, 08:00–20:00). The mice were fed experimental diets based on the American Institute of Nutrition (AIN)-93G formulation [28] and tap water *ad libitum*.

### 2.4. Single Oral Dose Toxicity Study

The single oral dose toxicity study (limit test) was conducted following the Organization for Economic Co-operation and Development (OECD) guidelines for the testing of chemicals (TG 423) [29]. Our previous study showed that administration of SCO-M and SCO-U at a dose of 2000 mg/kg body weight (BW)/day, which is the upper limit dose specified by the OECD Guideline for the Testing of Chemicals No. 474 [30], did not induce clinical signs or BW changes in ICR mice [22]. Therefore, 5000 mg/kg BW/day was set as the dose of SCO-M and SCO-U for the single oral dose toxicity study. Thirty-six mice (18 males and 18 females) were divided into three groups (SCO-M, SCO-U, and control) containing equal numbers of males and females. After fasting for 4 h, the test substances were administered to mice by using oral gavage. The control group was administrated an equal amount of olive oil instead of SCO-M or SCO-U. The mice were monitored for clinical signs (general behavior, motor activities, reflexes, cardiovascular signs, respiratory pattern, and changes in skin and fur texture) immediately and after 6 h of administration and thereafter once a day for 14 days. BW was measured every two days. After 14 days, overnight-fasted mice were sacrificed by cervical dislocation under isoflurane (Intervet K.K.; Osaka, Japan) anesthesia for macroscopic evaluation.

### 2.5. Repeated Oral Dose Toxicity Study for 28 Days

Seven groups of 8 male and female mice were fed diets containing 7% (w/w) oil for a period of 28 days. The control diet was based on the AIN93G formula [28] and contained 7% soybean oil. The experimental diets contained 6% soybean oil with 1% SCO-M (SCO-M 1% group) or SCO-U (SCO-U 1% group) and 2% soybean oil with 5% SCO-M (SCO-M 5% group) or SCO-U (SCO-U 5% group). In addition, the tuna oil diets contained 6% soybean oil with 1% tuna oil (TO 1% group) and 2% soybean oil with 5% tuna oil (TO 5% group). Because no toxicity was observed in the administration of SCO-M and SCO-U at a dose of 5000 mg/kg BW/day in the single oral dose toxicity study, the high dose of SCO-M and SCO-U was set at the 5%, which corresponded to 2.5–5 g/kg BW/day. The experimental diets were stored at −35 °C, thawed, and fed to the mice in a daily discard and top-up routine. Gross appearances behavior, and food intake of the mice were evaluated daily. After 28 days, overnight-fasted mice were euthanized under isoflurane anesthesia, and macroscopic evaluation was performed. Blood was collected from the inferior vena cava with and without using anti-coagulants, and serum was obtained by centrifugation (2000 × *g* for 15 min). Abdominal organs were quickly removed, weighed, rinsed with cold saline, and frozen in liquid nitrogen.

### 2.6. Repeated Oral Dose Toxicity Study for 13 Weeks

Four groups of 8 male and female mice were used for repeated oral dose toxicity study for 13 weeks (limit test). The Control diet was based on the AIN93G formula [28]. The experimental diets contained 2% soybean oil with 5% SCO-M, SCO-U, or tuna oil (SCO-M 5%, SCO-U 5%, or TO 5% groups, respectively). Other experimental and anatomical conditions were similar to the repeated oral dose toxicity study for 28 days.

### 2.7. Analysis of Serum Biochemical and Hematological Parameters

In repeated oral dose toxicity study for 28 days, total protein, albumin, albumin/globulin (A/G), aspartate aminotransferase (AST), alanine aminotransferase (ALT), serum urea nitrogen (SUN), total lipid, phospholipid (PL), triglyceride (TG), total cholesterol, high-density lipoprotein cholesterol (HDL-C), and non-HDL-C were measured using an Olympus AU5431 automatic analyzer (Olympus Co., Tokyo, Japan) by a commercial service (Japan Medical Laboratory, Osaka, Japan). On the other hand, in repeated oral dose toxicity study for 13 weeks, in addition to the above-mentioned parameters, the hematological parameters including red blood cell (RBC) count, white blood cell (WBC) count, platelet (PLT) count, hemoglobin (Hb), hematocrit (Ht), mean corpuscular volume (MCV), mean corpuscular hemoglobin (MCH), and mean corpuscular hemoglobin concentration (MCHC) were also measured.

### 2.8. Analysis of Thiobarbituric Acid Reactive Substances and α-Tocopherol

The levels of thiobarbituric acid reactive substances (TBARS) in liver were determined by spectrophotometric analysis of the reaction between thiobarbituric acid and malondialdehyde at 532 nm [31]. The α-Toc levels in liver were evaluated by HPLC as described above.

### 2.9. Statistical Analyses

Data are expressed as mean ± standard errors of the mean (SEM). The differences between multiple groups were evaluated using analysis of variance (ANOVA) and Tukey’s multiple comparison test. Statistical significance was set at *p* < 0.05. The analyses were performed using GraphPad Prism software version 7.0d (GraphPad Software, San Diego, CA, USA).

## 3. Results and Discussion

### 3.1. Lipid Composition of the Experimental Oils

The lipid composition of the experimental oils (soybean oil, SCO-M, SCO-U, and tuna oil) is summarized in Table 1. Compared to tuna oil, SCO-M and SCO-U showed low DHA content and high EPA and total omega-3 PUFA contents. SCO-M and SCO-U contained 99 and 175 mg/g of PL, respectively. SCO-M and SCO-U differ majorly in terms of PL and α-Toc contents. In addition, the amounts of Cd, As, mercury, dioxin, pesticide residues, polychlorobiphenyl, and DST in SCO-M and SCO-U is summarized in Table S1 and demonstrated that they satisfy the requirements for use as food ingredients.

### 3.2. Single Oral Dose Toxicity Study

To determine the toxicity and the lethal dose (LD_50_) of SCO-M and SCO-U, they were administered orally to male and female ICR mice at a single dose of 0 (control group) or 5000 mg/kg BW. No mortality, abnormalities in gross appearance and behavior, or clinical signs were observed in the mice during the experimental period. In addition, there were no significant differences in the BW of SCO-M and SCO-U groups compared to the control group (data not shown). Therefore, the LD_50_ of SCO-M and SCO-U was estimated to be more than 5000 mg/kg BW.

### 3.3. Repeated Oral Dose Toxicity Study for 28 Days

No mortality or abnormalities in gross appearance and behavior were observed in the mice during the experimental period. Growth parameters are summarized in Table S2. There were no significant differences in the initial BW, final BW, BW gain, food intake, and food efficiency among the male and female mice of different groups.

Macroscopic evaluation showed no abnormalities in the abdominal organs of all groups administered SCO-M or SCO-U (SCO-M 1%, SCO-M 5%, SCO-U 1%, and SCO-U 5% groups). Relative organ weights are summarized in Table S3. There were no significant differences in the relative weights of liver, kidney, heart, spleen, lung, gonadal organs (testis or ovary and uterus), brain, stomach, small intestine, large intestine, epididymal white adipose tissue (WAT) (in males only), perirenal WAT, and mesentery WAT among the male and female mice of different groups. As macroscopic evaluation and relative organ weights did not reveal any abnormalities, no histopathological evaluations were performed.

The serum biochemical parameters are summarized in Table 2. As compared to control group, SCO-M and SCO-U groups showed no significant differences in the levels of total protein, albumin, and A/G (parameters for nutritional index), AST and ALT (parameters for liver function test), and SUN (parameter for kidney function test). The serum lipid parameters including total lipid, PL, total cholesterol, HDL-C, and non-HDL-C in both male and female mice of the SCO-M 5% and SCO-U 5% groups were significantly lower than that in the control group mice. The serum TG level of SCO-M 5% and SCO-U 5% groups was also lower than that of control group; however, the difference was not significant. In addition, the TO 5% group also showed a decrease in the levels of all serum lipid parameters. Previous studies suggest that intake of fish oil decreases the levels of serum TG and total cholesterol due to inhibition of lipogenesis through decrease in the levels of sterol regulatory element-binding proteins and stimulation of lipolysis via peroxisome proliferator-activator receptor α activation in liver [32,33]. Therefore, the reduction in the levels of different serum lipid parameters due to SCO-M and SCO-U intake does not seem to have an adverse effect.

EPA and DHA are easily oxidized as compared to LA and ALA because they have many methylene groups [34]. Therefore, dietary SCO-M, SCO-U, and tuna oil, which are rich in EPA and DHA, may promote lipid peroxidation in the body. To evaluate the degree of lipid peroxidation, TBARS and α-Toc levels in liver were analyzed. Liver TBARS and α-Toc levels in male and female mice are summarized in Table S4. There were no significant differences in liver TBARS levels among the male and female mice of different groups. On the contrary, male and female mice of SCO-M 5% and TO 5% groups showed lower α-Toc level in the liver as compared to control group mice; however, the difference was not significant. α-Toc is a well-known lipophilic antioxidant that can prevent lipid peroxidation [35]. These results suggest that the oxidation of α-Toc might precede the oxidation of EPA and DHA. Intake of tuna oil, which is already available commercially, also reduces liver α-Toc levels; therefore, it was not an undesirable specific effect of SCO-M and SCO-U. In the SCO-U 5% group, the liver α-Toc levels did not decrease as compared to the control group. The higher α-Toc content of SCO-U as compared to SCO-M and tuna oil may have prevented the decrease in liver α-Toc level. Therefore, increasing the amount of α-Toc added during the production process of SCO may alleviate the decrease in liver α-Toc level.

The decrease in serum lipid parameters observed in the SCO-M 5% and SCO-U 5% groups can be attributed to the effect of EPA and DHA contained in SCO-M and SCO-U because there was a decrease in serum lipid parameters of the TO 5% group also. Other parameters in the SCO-M 5% and SCO-U 5% groups were not significantly different from that in the control group and were considered within the normal range from the view point of biological levels. In addition, the minimum daily intakes of SCO-M and SCO-U during the experimental period were 3701 and 4423 mg/kg BW in males and 3829 and 4214 mg/kg BW in females, respectively. These results indicate that the dietary supplementation of 5% SCO-M or SCO-U had no toxic effects in ICR mice during the experimental period of 28 days.

### 3.4. Repeated Oral Dose Toxicity Study for 13 Weeks

Mice fed 5% SCO-M and 5% SCO-U diets did not show mortality, abnormalities in gross appearance and behavior, nor clinical signs in repeated oral dose toxicity study for 28 days. Therefore, in the repeated oral dose toxicity study for 13 weeks (limit test), 5% SCO-M and 5% SCO-U diets were used for evaluating the toxicities of SCO-M and SCO-U. No mortality or abnormalities in gross appearance of the mice were observed during the experimental period. The growth parameters are summarized in Table S5. There were no significant differences in the initial BW, final BW, BW gain, food intake, and food efficiency among the male and female mice of different groups.

Relative organ weights are summarized in Table 3. Macroscopic evaluation showed no abnormalities in the abdominal organs of the SCO-M 5% and SCO-U 5% group mice. A previous study reported the microscopic evidence of yellow fat disease in animals fed fish oil, mainly when dietary vitamin E content was low [36]. Yellow fat disease was not observed in any of the mice used in this study. There were no significant differences in the relative weights of liver, kidney, heart, spleen, lung, testis, brain, stomach, small intestine, large intestine, mesentery WAT, perirenal WAT, and epididymal WAT among the male mice of different groups. On the contrary, the relative weight of mesentery WAT in females of SCO-U 5% group was significantly higher compared to that in female mice of control group. This may be due to the higher food intake and weight gain tendency of the SCO 5% group female mice. Therefore, the increased relative weight of mesentery WAT in the females of SCO-U 5% group was not considered as a severe toxicological problem. Other organ weights were not significantly different among the females of different groups. As macroscopic evaluation and relative organ weights did not reveal any abnormalities, no histopathological evaluations were performed.

The biochemical and hematological parameters are summarized in Table 4. No significant differences were observed in the biochemical parameters including total protein, albumin, A/G, AST, ALT, and SUN levels in the SCO-M 5% and SCO-U 5% groups compared to the control group. In addition, the hematological parameters including RBC count, WBC count, PLT count, Hb, Ht, MCV, MCH, and MCHC in the SCO-M 5% and SCO-U 5% groups were also not significantly different compared to that in the control group. The serum lipid parameters including total lipid, PL, total cholesterol, HDL-C, and non-HDL-C levels were significantly decreased in the male mice of SCO-M 5% and SCO-U 5% groups as compared to the male mice of the control group. In addition, PL and Non-HDL-C levels were significantly lower in the female mice of SCO-M 5% and SCO-U 5% groups as compared to the control group female mice. Decrease in serum lipid parameters was also observed in the TO 5% group. Therefore, the reduction in serum lipid parameters due to the intake of SCO-M and SCO-U for 13 weeks had no adverse effects.

Liver TBARS and α-Toc levels in male and female mice are summarized in Table 5. No significant differences in liver TBARS levels were observed among the male and female mice of different groups. On the contrary, liver α-Toc levels in the female mice of SCO-M 5%, SCO-U 5%, and TO 5% groups were lower as compared to control group female mice. In addition, liver α-Toc levels in the male mice of SCO-M 5% and TO 5% groups were lower as compared to control group male mice (*p* = 0.15 and 0.17, respectively). As in the repeated oral dose toxicity study for 28 days, the intake of SCO-M and SCO-U did not affect liver TBARS levels, but it reduced liver α-Toc levels. As mentioned above, liver α-Toc might inhibit the oxidation of PUFA, including EPA and DHA, by its own oxidation. Although the SCO-M 5% and SCO-U 5% groups showed a decrease in liver α-Toc levels, there was no increase in liver TBARS levels; therefore, this is not a serious toxicological problem.

This repeated oral dose toxicity study for 13 weeks in mice using SCO-M and SCO-U at a dose of 5% in the diets demonstrated that SCO-M and SCO-U did not have any toxicologically significant adverse effects and were safe under the experimental condition of this study. The minimum daily intakes of SCO-M and SCO-U during the experimental period were 3,683 and 3,826 mg/kg BW in males and 3,840 and 3,801 mg/kg BW in females, respectively. However, as only one particular dose of SCO-M and SCO-U was used in this study, no definitive statement on the no-observed-adverse-effect-level can be made, which is a limitation of this study.

Another limitation of this study is that histopathological evaluations were not conducted in the 28-day and 13-week repeated oral dose toxicity studies as macroscopic evaluation and relative organ weights did not reveal any abnormalities. However, histopathological evaluations are often carried out in toxicity studies. Therefore, histopathological studies are required to assure the further safety of SCO-M and SCO-U.

## 4. Conclusions

In the single oral dose toxicity study using mice, the LD_50_ of SCO-M and SCO-U was estimated to be greater than 5,000 mg/kg BW. In the 28-day and 13-week repeated oral dose toxicity studies, intake of SCO-M or SCO-U at a concentration of 5% (w/w) in the diets had no significant toxicological effects. SCO-M and SCO-U were well-tolerated at the tested dietary levels as evidenced by the absence of major treatment-related changes in general condition and appearance, growth, and hematological and clinical chemistry parameters of the mice. These results suggest that SCO-M and SCO-U have no adverse toxicological and clinical chemistry effects in mice when administered at a dose of 5% in the diets of mice for 28 days, as well as 13 weeks. When SCO-M or SCO-U at a concentration of 5% (*w/w*) in the diets converted to human equivalent doses, the dose level provides safety margin of about 12- to 24-fold with the recommended fish oil supplement level of 1 g/day. This supports the safe availability of SCO-M and SCO-U as a supplement. In addition, both SCO-M and SCO-U have a toxicity profile similar to other omega-3 PUFA sources [37,38,39]. Thus, our study demonstrates that SCO-M and SCO-U are safe in terms of acute and sub-acute toxicities under the present experimental conditions and have the potential to be used as alternative sources of omega-3 PUFA.

## Figures and Tables

**Table 1:** Lipid composition of the experimental oils.

		Soybean Oil	SCO		Tuna Oil
			SCO-M ^1^	SCO-U ^2^	
Fatty acid composition (wt%)					
	C14:0	0.1	5.1	4.5	2.7
	C16:0	11.6	16.3	16.6	19.2
	C16:1n-7	0.1	11.5	9.8	5.8
	C18:0	1.6	2.6	3.8	1.2
	C18:1n-9	23.2	2.8	2.9	24.3
	C18:1n-7	1.8	5.9	5.4	2.2
	C18:2n-6	49.2	1.8	1.2	1.3
	C18:3n-3	5.5	0.8	0.9	0.4
	C20:4n-6 (ARA)	-	3.6	1.5	2.2
	C20:5n-3 (EPA)	-	29.4	29.6	5.7
	C22:6n-3 (DHA)	-	10.5	10.8	23.9
	Others	7.0	9.7	13	11.4
Phospholipid (mg/g)		N.D.	99	175	1
Cholesterol (mg/g)		0.1	5.6	7.6	1.5
α-Toc (µg/g)		-	1.28	6.87	0.46
PV (meq/kg)		6.7	6.4	3.5	2.9
AV (mg/g)		0.1	6.7	21.6	1.5

^1^ SCO prepared from the scallop internal organs from Mutsu bay area (Aomori, Japan). ^2^ SCO prepared from the scallop internal organs from Uchiura bay area (Hokkaido, Japan). ARA, arachidonic acid; AV, acid value; DHA, docosahexaenoic acid; EPA, eicosapentaenoic acid; N.D., not detected; PV, peroxide value; Toc, tocopherol; SCO, scallop oil.

**Table 2:** Serum biochemical parameters of mice administered scallop oil (SCO) for 28 days.

		Control			SCO-M						SCO-U						TO					
					1%			5%			1%			5%			1%			5%		
Male																						
	Total protein (g/dL)	5.13	±	0.06	5.15	±	0.06	5.13	±	0.06	5.00	±	0.07	5.03	±	0.08	4.90	±	0.05	5.15	±	0.09
	Albumin (g/dL)	2.98	±	0.03 ^a^	2.90	±	0.06 ^ab^	2.86	±	0.03 ^ab^	2.81	±	0.05 ^ab^	2.86	±	0.04 ^ab^	2.74	±	0.03 ^b^	2.94	±	0.07 ^ab^
	A/G	1.38	±	0.02	1.29	±	0.05	1.27	±	0.02	1.29	±	0.03	1.32	±	0.02	1.27	±	0.03	1.33	±	0.03
	AST (IU/L)	51.6	±	5.1	77.9	±	14.0	47.0	±	2.5	68.1	±	11.6	65.6	±	10.1	50.0	±	4.9	53.4	±	4.6
	ALT (IU/L)	40.6	±	7.4	66.1	±	18.5	44.0	±	5.6	69.8	±	19.1	89.0	±	21.7	35.5	±	5.3	61.1	±	15.4
	SUN (mg/dL)	22.9	±	1.0	20.3	±	1.4	23.0	±	0.9	23.1	±	1.9	22.9	±	1.8	23.0	±	1.8	21.0	±	0.4
	Total lipid (mg/dL)	500	±	26 ^a^	437	±	43 ^ab^	322	±	13 ^b^	472	±	44 ^a^	320	±	36 ^b^	459	±	21 ^ab^	349	±	28 ^b^
	PL (mg/dL)	251	±	8 ^a^	227	±	19 ^a^	159	±	4 ^b^	204	±	13 ^a^	155	±	11 ^b^	219	±	11 ^a^	154	±	7 ^b^
	TG (mg/dL)	129	±	15	103	±	15	78	±	8	149	±	26	80	±	17	124	±	12	101	±	18
	Total cholesterol (mg/dL)	135	±	5 ^a^	123	±	10 ^ab^	90	±	3 ^b^	111	±	11 ^ab^	88	±	8 ^b^	120	±	8 ^ab^	88	±	5 ^b^
	HDL-C (mg/dL)	106	±	3 ^a^	98	±	8 ^a^	75	±	2 ^b^	87	±	7 ^ab^	74	±	6 ^b^	95	±	6 ^ab^	70	±	4 ^b^
	Non-HDL-C (mg/dL)	28.8	±	2.6 ^a^	24.9	±	2.8 ^ab^	14.3	±	1.0 ^b^	23.5	±	3.6 ^ab^	13.6	±	2.1 ^b^	25.6	±	2.5 ^a^	17.4	±	1.7 ^b^
Female																						
	Total protein (g/dL)	4.96	±	0.05	4.85	±	0.08	4.91	±	0.07	4.98	±	0.08	4.79	±	0.04	4.84	±	0.06	5.03	±	0.05
	Albumin (g/dL)	3.11	±	0.04	3.06	±	0.05	3.04	±	0.05	3.10	±	0.05	3.00	±	0.02	3.04	±	0.05	3.16	±	0.05
	A/G	1.68	±	0.03	1.71	±	0.03	1.62	±	0.03	1.65	±	0.02	1.68	±	0.02	1.69	±	0.03	1.70	±	0.04
	AST (IU/L)	45.7	±	2.0	48.8	±	4.8	48.9	±	3.1	50.9	±	3.6	49.1	±	3.7	50.0	±	4.7	51.3	±	3.5
	ALT (IU/L)	29.9	±	10.6	19.5	±	3.0	27.0	±	3.0	24.6	±	4.2	22.3	±	3.1	18.7	±	1.2	26.3	±	4.3
	SUN (mg/dL)	20.4	±	1.0	22.3	±	1.1	21.5	±	1.1	20.9	±	0.5	22.0	±	1.4	19.3	±	1.1	21.0	±	1.0
	Total lipid (mg/dL)	392	±	33 ^a^	348	±	16 ^ab^	258	±	7 ^b^	378	±	26 ^a^	246	±	17 ^b^	331	±	19 ^ab^	271	±	20 ^b^
	PL (mg/dL)	183	±	8 ^a^	170	±	7 ^a^	122	±	3 ^b^	175	±	7 ^a^	119	±	6 ^b^	168	±	7 ^ab^	129	±	8 ^b^
	TG (mg/dL)	121	±	16 ^a^	102	±	9 ^ab^	70	±	4 ^b^	118	±	15 ^a^	68	±	6 ^b^	85	±	10 ^ab^	73	±	6 ^b^
	Total cholesterol (mg/dL)	94	±	6 ^a^	87	±	7 ^ab^	68	±	3 ^b^	90	±	4 ^ab^	63	±	5 ^b^	89	±	4 ^ab^	71	±	6 ^ab^
	HDL-C (mg/dL)	69	±	4 ^a^	65	±	4 ^ab^	53	±	2 ^b^	68	±	2 ^a^	52	±	3 ^b^	69	±	3 ^a^	54	±	4 ^b^
	Non-HDL-C (mg/dL)	25.1	±	2.2 ^a^	21.6	±	2.4 ^ab^	14.6	±	0.9 ^bc^	21.5	±	1.5 ^ab^	11.7	±	1.7 ^c^	20.0	±	1.3 ^ab^	17.0	±	2.1 ^bc^

Data are represented as mean ± standard errors of the mean (SEM) (n = 8). Values in the same row having different superscripts are significantly different at *p* < 0.05 using Tukey’s multiple comparison test. AST, aspartate aminotransferase; ALT, alanine aminotransferase; A/G, albumin/globulin; HDL-C, high-density lipoprotein cholesterol; PL, phospholipid; SUN, serum urea nitrogen; SCO, scallop oil; SCO-M, SCO from Mutsu bay, Aomori, Japan; SCO-U, SCO from Uchiura bay, Hokkaido, Japan; TO, tuna oil; TG, triglyceride.

**Table 3:** Organ weights of mice administered with scallop oil for 13 weeks.

		Control			SCO-M 5%			SCO-U 5%			TO 5%		
		g/100 g BW											
Male													
	Liver	3.82	±	0.15	4.43	±	0.09	4.27	±	0.24	4.17	±	0.15
	Kidney	1.20	±	0.05	1.11	±	0.07	1.11	±	0.04	1.19	±	0.04
	Heart	0.55	±	0.03	0.49	±	0.03	0.47	±	0.02	0.51	±	0.03
	Spleen	0.41	±	0.02	0.42	±	0.03	0.46	±	0.04	0.47	±	0.03
	Lung	0.49	±	0.03	0.51	±	0.04	0.50	±	0.02	0.55	±	0.04
	Testis	0.57	±	0.03	0.43	±	0.03	0.54	±	0.04	0.59	±	0.03
	Brain	0.99	±	0.05	0.89	±	0.03	0.91	±	0.03	1.01	±	0.05
	Stomach	1.40	±	0.19	1.23	±	0.21	1.42	±	0.16	1.72	±	0.26
	Small intestine	3.15	±	0.15	3.08	±	0.14	3.26	±	0.12	3.14	±	0.12
	Large intestine	0.49	±	0.04	0.52	±	0.04	0.63	±	0.10	0.46	±	0.21
	Mesentery WAT	4.14	±	0.48	4.00	±	0.30	3.91	±	0.20	3.01	±	0.33
	Perirenal WAT	1.24	±	0.16	1.48	±	0.17	1.34	±	0.10	1.03	±	0.20
	Epididymal WAT	1.92	±	0.16	2.41	±	0.15	2.23	±	0.19	1.89	±	0.15
Female													
	Liver	4.03	±	0.12	4.05	±	0.18	3.69	±	0.21	4.16	±	0.16
	Kidney	1.24	±	0.07	0.97	±	0.09	1.05	±	0.06	1.18	±	0.05
	Heart	0.60	±	0.04	0.51	±	0.03	0.54	±	0.04	0.55	±	0.03
	Spleen	0.58	±	0.03	0.60	±	0.05	0.54	±	0.05	0.64	±	0.06
	Lung	0.81	±	0.04	0.78	±	0.07	0.69	±	0.06	0.75	±	0.04
	Ovary	0.27	±	0.01	0.22	±	0.02	0.25	±	0.02	0.25	±	0.02
	Uterus	0.43	±	0.04	0.36	±	0.07	0.31	±	0.04	0.39	±	0.05
	Brain	1.59	±	0.06	1.28	±	0.07	1.26	±	0.11	1.39	±	0.06
	Stomach	2.00	±	0.11	1.82	±	0.16	2.13	±	0.35	2.17	±	0.27
	Small intestine	4.41	±	0.22	3.56	±	0.23	3.70	±	0.28	4.01	±	0.20
	Large intestine	0.91	±	0.09	0.87	±	0.05	0.75	±	0.06	0.81	±	0.03
	Mesentery WAT	0.68	±	0.08 ^b^	1.35	±	0.22 ^ab^	1.69	±	0.39 ^a^	1.03	±	0.18 ^ab^
	Perirenal WAT	1.62	±	0.07	2.58	±	0.33	2.78	±	0.49	2.29	±	0.24

Data are represented as mean ± SEM (n = 8). Values in the same row having different superscripts are significantly different at *p* < 0.05 using Tukey’s multiple comparison test. BW, body weight; SCO, scallop oil; SCO-M, SCO from Mutsu bay, Aomori, Japan; SCO-U, SCO from Uchiura bay, Hokkaido, Japan; TO, tuna oil; WAT, white adipose tissue.

**Table 4:** The biochemical and hematological parameters of mice administered with scallop oil for 13 weeks.

		Control			SCO-M 5%			SCO-U 5%			TO 5%		
Male													
	Total protein (g/dL)	2.7	±	0.0	2.6	±	0.1	2.6	±	0.1	2.6	±	0.0
	Albumin (g/dL)	1.4	±	0.0	1.3	±	0.0	1.3	±	0.1	1.3	±	0.0
	A/G	1.0	±	0.0	1.0	±	0.0	1.0	±	0.1	1.0	±	0.0
	AST (IU/L)	11.9	±	0.7	12.3	±	1.1	11.6	±	0.9	10.1	±	0.5
	ALT (IU/L)	51.4	±	12.5	35.1	±	2.6	28.8	±	2.1	87.9	±	35.8
	SUN (mg/dL)	26.3	±	4.9	30.7	±	4.7	22.0	±	3.5	50.4	±	25.0
	Total lipid (mg/dL)	176	±	18 ^a^	106	±	6 ^b^	110	±	14 ^b^	118	±	14 ^b^
	PL (mg/dL)	109	±	9 ^a^	61	±	3 ^b^	65	±	8 ^b^	68	±	7 ^b^
	TG (mg/dL)	26	±	5	19	±	1	17	±	3	21	±	4
	Total cholesterol (mg/dL)	59	±	6 ^a^	34	±	2 ^b^	36	±	5 ^b^	38	±	5 ^b^
	HDL-C (mg/dL)	50	±	5 ^a^	31	±	2 ^b^	33	±	4 ^b^	33	±	4 ^b^
	Non-HDL-C (mg/dL)	9	±	2 ^a^	3	±	0 ^b^	4	±	1 ^b^	5	±	1 ^b^
	RBC (10^4^ cell/µL)	817	±	7	836	±	15	822	±	16	751	±	32
	WBC (cell/µL)	3157	±	868	4120	±	421	3133	±	414	3267	±	230
	PLT (10^4^ cell/µL)	13.7	±	3.0	20.3	±	3.7	19.5	±	0.7	19.4	±	3.2
	Hb (g/dL)	13.5	±	0.1 ^ab^	14.3	±	0.4 ^ab^	14.2	±	0.1 ^a^	12.6	±	0.4 ^b^
	Ht (%)	42.6	±	0.5	45.0	±	1.0	44.0	±	1.1	41.4	±	0.9
	MCV (fL)	52.1	±	0.3	53.8	±	0.8	53.7	±	1.0	55.7	±	1.3
	MCH (pg)	16.5	±	0.2	17.0	±	0.2	17.2	±	0.3	16.8	±	0.2
	MCHC (g/dL)	31.7	±	0.2	31.7	±	0.4	32.3	±	0.7	30.3	±	0.3
Female													
	Total protein (g/dL)	2.5	±	0.0	2.5	±	0.0	2.6	±	0.0	2.5	±	0.0
	Albumin (g/dL)	1.4	±	0.0	1.4	±	0.0	1.4	±	0.0	1.4	±	0.0
	A/G	1.3	±	0.0	1.2	±	0.0	1.2	±	0.0	1.2	±	0.0
	AST (IU/L)	11.6	±	0.8	13.5	±	0.5	11.9	±	0.7	11.4	±	0.7
	ALT (IU/L)	31.3	±	2.6	30.4	±	3.6	32.6	±	3.5	34.8	±	3.8
	SUN (mg/dL)	10.5	±	1.4	13.5	±	2.1	17.8	±	5.9	14.4	±	2.0
	Total lipid (mg/dL)	128	±	9 ^a^	89	±	7 ^b^	101	±	13 ^ab^	93	±	9 ^ab^
	PL (mg/dL)	76	±	4 ^a^	53	±	3 ^b^	57	±	6 ^b^	52	±	4 ^b^
	TG (mg/dL)	30	±	3	20	±	3	22	±	3	23	±	3
	Total cholesterol (mg/dL)	37	±	2	26	±	2	30	±	4	26	±	3
	HDL-C (mg/dL)	28	±	2	23	±	2	24	±	3	21	±	2
	Non-HDL-C (mg/dL)	8	±	1 ^a^	3	±	0 ^b^	5	±	1 ^b^	4	±	1 ^b^
	RBC (10^4^ cell/µL)	878	±	16	847	±	13	847	±	18	871	±	25
	WBC (cell/µL)	3125	±	373	4138	±	650	3850	±	320	3586	±	744
	PLT (10^4^ cell/µL)	18.8	±	5.2	22.3	±	6.8	17.7	±	3.4	19.7	±	5.0
	Hb (g/dL)	14.3	±	0.1	14.0	±	0.2	14.1	±	0.2	14.2	±	0.3
	Ht (%)	44.4	±	0.3	43.7	±	0.5	44.8	±	0.7	44.2	±	1.0
	MCV (fL)	50.8	±	0.7	51.8	±	0.5	53.1	±	0.7	50.9	±	0.5
	MCH (pg)	16.3	±	0.2	16.5	±	0.2	16.7	±	0.2	16.4	±	0.2
	MCHC (g/dL)	32.1	±	0.1	32.0	±	0.4	31.5	±	0.2	32.2	±	0.2

Data are represented as mean ± SEM (n = 8). Values in the same row having different superscripts are significantly different at *p* < 0.05 using Tukey’s multiple comparison test. AST, aspartate aminotransferase; ALT, alanine aminotransferase; A/G, albumin/globulin; Hb, hemoglobin, Ht, hematocrit; HDL-C, high-density lipoprotein cholesterol; MCH, mean corpuscular hemoglobin; MCHC, mean corpuscular hemoglobin concentration; MCV, mean corpuscular volume; PL, phospholipid; PLT, platelet; RBC, red blood cell; SUN, serum urea nitrogen; SCO, scallop oil; SCO-M, SCO from Mutsu bay, Aomori, Japan; SCO-U, SCO from Uchiura bay, Hokkaido, Japan; TO, tuna oil; TG, triglyceride; WBC, white blood cell.

**Table 5:** Liver thiobarbituric acid reactive substances (TBARS) and α-tocopherol (α-Toc) levels in mice administered with scallop oil for 13 weeks.

		Control			SCO-M 5%			SCO-U 5%			TO 5%		
Male													
	TBARS (μmol/g)	98	±	5	115	±	18	111	±	7	119	±	6
	α-Toc (μg/g)	104.6	±	17.0	53.9	±	6.2	79.9	±	23.0	57.6	±	10.2
Female													
	TBARS (μmol/g)	88	±	8	87	±	13	94	±	6	95	±	8
	α-Toc (μg/g)	63.5	±	7.7 ^a^	37.1	±	2.9 ^b^	37.0	±	3.0 ^b^	37.1	±	7.7 ^b^

Data are represented as mean ± SEM (n = 8). Values in the same row having different superscripts are significantly different at *p* < 0.05 using Tukey’s multiple comparison test. SCO, scallop oil; SCO-M, SCO from Mutsu bay, Aomori, Japan; SCO-U, SCO from Uchiura bay, Hokkaido, Japan; TBARS, thiobarbituric acid reactive substances; α-Toc, α-tocopherol; TO, tuna oil.

## Supplementary Materials

The following are available online at https://www.mdpi.com/2304-8158/9/6/691/s1, Table S1: Harmful substance contents of regulated values and the experimental oils, Table S2: Growth parameters of mice administered scallop oil for 28 days, Table S3: Organ weights of mice administered scallop oil for 28 days, Table S4: Liver thiobarbituric acid reactive substances (TBARS) and α-tocopherol (α-Toc) levels in mice administered scallop oil for 28 days, Table S5: Growth parameters of mice administered scallop oil for 13 weeks.
