# Supplementary material for: Single and Repeated Dose 28-Day and 13-Week Toxicity Studies of Oil Prepared from the Internal Organs of the Japanese Giant Scallop (Patinopecten yessoensis) in Mice
Source: Foods. 2020 May 27;9(6):691. doi: 10.3390/foods9060691 (PMC7353646; doi:10.3390/foods9060691)
Supplement: Supplementary file 1 [file foods-09-00691-s001.pdf]

Table S1. Harmful substance contents of regulated values and the experimental oils.

	Unit	Regulated values				Test substances	
		FAO/WHO Codex Alimentarius	Food Chemical Codex	European Union E322	Food Sanitation Act in Japan	SCO-M[1]	SCO-U[2]
**Cadmium**	mg/kg	< 2	-	-	-	0.03	0.01
Mercury	ppm	-	< 1	-	< 0.4	N.D. (< 0.01)	N.D. (< 0.01)
Arsenic	ppm	< 3	< 3	-	-	1.22	0.94
Dioxin	pg-TEQ/kg	-	-	< 2	-	0.04	0.04
Polychlorobiphenyl	ppm	-	-	< 6	< 3	N.D. (< 0.1)	N.D. (< 0.1)
Pesticide residues	ppm	< 0.1	< 6	-	< 0.1	N.D. (< 0.01)	N.D. (< 0.01)
Diarrhetic shellfish toxin	mg okadaic acid Eq/kg	< 0.16 (shellfish)	-	-	-	0.09	0.04

[1] SCO prepared from the scallop internal organs from Mutsu bay area (Aomori, Japan).[2] SCO prepared from the scallop internal organs from Uchiura bay area (Hokkaido, Japan). N.D., not detected; SCO, scallop oil; TEQ, toxic equivalent.

Table S2. Growth parameters of mice administered scallop oil (SCO) for 28 days.

	**Control**	**SCO-M**		**SCO-U**		TO	
		**1%**	**5%**	**1%**	**5%**	1%	5%
**Male**							
Initial BW (g)	25.7 ± 0.3	26.1 ± 0.3	25.4 ± 0.3	26.3 ± 0.3	25.5 ± 0.3	25.5 ± 0.3	25.9 ± 0.3
Final BW (g)	40.4 ± 2.1	41.8 ± 1.4	41.7 ± 0.9	42.5 ± 1.0	41.8 ± 1.2	40.5 ± 2.0	39.5 ± 1.2
BW gain (g/day)	0.49 ± 0.06	0.52 ± 0.05	0.54 ± 0.04	0.54 ± 0.03	0.54 ± 0.04	0.50 ± 0.06	0.45 ± 0.04
Food intake (g/day)	3.9 ± 0.1	4.1 ± 0.1	4.0 ± 0.1	4.0 ± 0.1	4.0 ± 0.1	3.9 ± 0.1	3.8 ± 0.1
Food efficiency (g/g)[1]	0.13 ± 0.02	0.13 ± 0.01	0.14 ± 0.01	0.14 ± 0.01	0.14 ± 0.01	0.13 ± 0.02	0.12 ± 0.01
Female							
Initial BW (g)	21.5 ± 0.3	21.9 ± 0.3	21.8 ± 0.3	21.4 ± 0.3	21.4 ± 0.3	21.9 ± 0.4	21.5 ± 0.3
Final BW (g)	32.5 ± 0.8	34.0 ± 0.7	34.4 ± 1.0	32.7 ± 0.8	31.2 ± 0.9	31.6 ± 0.7	33.0 ± 1.0
BW gain (g/day)	0.36 ± 0.03	0.39 ± 0.02	0.41 ± 0.04	0.37 ± 0.03	0.32 ± 0.03	0.31 ± 0.03	0.37 ± 0.03
Food intake (g/day)	3.2 ± 0.0	3.3 ± 0.1	3.3 ± 0.1	3.3 ± 0.1	3.0 ± 0.1	3.1 ± 0.1	3.3 ± 0.1
Food efficiency (g/g) [1]	0.11 ± 0.01	0.12 ± 0.01	0.12 ± 0.01	0.11 ± 0.01	0.11 ± 0.01	0.10 ± 0.01	0.11 ± 0.01

Data are represented as mean ± standard errors of the mean (SEM) (n = 8). [1] Food efficiency (g/g) = BW gain (g/day) / food intake (g/day). BW, body weight; SCO, scallop oil; TO, tuna oil.

Table S3. Organs weights of mice administered scallop oil (SCO) for 28 days.

	Control	SCO-M		SCO-U		TO	
		1%	5%	1%	5%	1%	5%
				g/100 g BW			
**Male**							
Liver	4.75 ± 0.17	4.31 ± 0.20	4.50 ± 0.10	4.60 ± 0.15	4.37 ± 0.14	4.28 ± 0.13	4.45 ± 0.13
Kidney	2.04 ± 0.06	2.06 ± 0.08	2.00 ± 0.05	2.07 ± 0.08	1.89 ± 0.06	2.13 ± 0.05	2.14 ± 0.07
Heart	1.02 ± 0.11	1.05 ± 0.03	1.01 ± 0.02	1.13 ± 0.16	0.98 ± 0.03	1.05 ± 0.05	1.00 ± 0.02
Spleen	0.31 ± 0.01	0.34 ± 0.03	0.36 ± 0.02	0.33 ± 0.02	0.35 ± 0.02	0.35 ± 0.01	0.35 ± 0.01
Lung	1.07 ± 0.03	1.05 ± 0.03	1.06 ± 0.02	1.00 ± 0.04	1.02 ± 0.02	1.03 ± 0.03	1.06 ± 0.03
Testis	1.36 ± 0.06	1.30 ± 0.05	1.23 ± 0.03	1.23 ± 0.04	1.26 ± 0.05	1.30 ± 0.07	1.34 ± 0.06
Brain	2.02 ± 0.10	1.97 ± 0.06	1.94 ± 0.03	1.93 ± 0.05	1.97 ± 0.07	2.01 ± 0.10	2.08 ± 0.07
Stomach	1.75 ± 0.26	1.47 ± 0.16	1.45 ± 0.22	1.26 ± 0.14	1.38 ± 0.21	1.65 ± 0.23	1.75 ± 0.24
Small intestine	4.24 ± 0.30	3.72 ± 0.35	4.07 ± 0.30	3.59 ± 0.26	3.85 ± 0.39	3.87 ± 0.20	4.15 ± 0.30
Large intestine	1.09 ± 0.08	1.07 ± 0.11	1.21 ± 0.11	1.04 ± 0.11	1.32 ± 0.14	0.94 ± 0.11	0.98 ± 0.10
Epididymal WAT	3.74 ± 0.38	3.93 ± 0.33	3.77 ± 0.21	4.14 ± 0.23	3.87 ± 0.25	4.03 ± 0.29	3.72 ± 0.20
Perirenal WAT	1.48 ± 0.14	1.56 ± 0.16	1.50 ± 0.22	1.70 ± 0.16	1.70 ± 0.09	1.65 ± 0.10	1.43 ± 0.10
Mesentery WAT	2.03 ± 0.19	1.95 ± 0.20	2.10 ± 0.14	2.29 ± 0.18	2.13 ± 0.17	1.99 ± 0.16	1.95 ± 0.12
Female							
Liver	4.68 ± 0.26	4.79 ± 0.18	4.91 ± 0.15	4.97 ± 0.19	4.68 ± 0.18	4.78 ± 0.15	4.76 ± 0.10
Kidney	1.18 ± 0.02	1.19 ± 0.03	1.22 ± 0.05	1.17 ± 0.04	1.19 ± 0.05	1.12 ± 0.06	1.16 ± 0.02
Heart	0.70 ± 0.27	0.52 ± 0.04	0.56 ± 0.05	0.59 ± 0.04	0.58 ± 0.04	0.47 ± 0.04	0.49 ± 0.02
Spleen	0.51 ± 0.05	0.55 ± 0.04	0.63 ± 0.07	0.51 ± 0.04	0.56 ± 0.04	0.50 ± 0.04	0.57 ± 0.04
Lung	0.91 ± 0.24	0.70 ± 0.03	0.71 ± 0.03	0.68 ± 0.03	0.69 ± 0.02	0.69 ± 0.03	0.71 ± 0.04
Ovary	0.11 ± 0.01	0.11 ± 0.02	0.12 ± 0.01	0.10 ± 0.01	0.09 ± 0.02	0.12 ± 0.03	0.10 ± 0.01
Uterus	0.63 ± 0.15	0.67 ± 0.09	0.46 ± 0.04	0.50 ± 0.06	0.53 ± 0.09	0.63 ± 0.09	0.45 ± 0.05
Brain	1.58 ± 0.07	1.50 ± 0.06	1.47 ± 0.06	1.56 ± 0.03	1.67 ± 0.05	1.58 ± 0.05	1.50 ± 0.05
Stomach	2.14 ± 0.29	3.08 ± 0.42	2.98 ± 0.47	2.70 ± 0.43	2.42 ± 0.21	2.60 ± 0.28	2.61 ± 0.39
Small intestine	4.51 ± 0.18	4.76 ± 0.39	4.62 ± 0.19	4.42 ± 0.20	4.51 ± 0.17	4.60 ± 0.15	4.72 ± 0.22

Table S3. *Continued*

	Control	SCO-M		SCO-U		TO	
		1%	5%	1%	5%	1%	5%
				g/100 g BW			
**Large intestine**	**1.03 ± 0.09**	**1.02 ± 0.07**	**1.07 ± 0.09**	**1.06** ± 0.13	1.05 ± 0.13	1.04 ± 0.11	0.92 ± 0.06
Perirenal WAT	0.83 ± 0.15	0.73 ± 0.09	0.70 ± 0.09	0.89 ± 0.09	0.53 ± 0.12	0.72 ± 0.15	0.98 ± 0.15
Mesentery WAT	1.91 ± 0.08	2.04 ± 0.18	1.80 ± 0.10	1.78 ± 0.08	1.75 ± 0.11	1.73 ± 0.11	2.06 ± 0.10

Data are represented as mean ± SEM (n = 8). BW, body weight; SCO, scallop oil; SCO-M, SCO from Mutsu bay, Aomori, Japan; SCO-U, SCO from Uchiura bay, Hokkaido, Japan; TO, tuna oil; WAT, white adipose tissue.

Table S4. Liver thiobarbituric acid reactive substances (TBARS) and α-tocopherol (α-Toc) levels in mice administered scallop oil (SCO) for 28 days.

	Control	SCO-M		SCO-U		TO	
		1%	5%	1%	5%	1%	5%
**Male**							
TBARS (μmol/g)	26.5 ± 4.5	29.7 ± 3.1	32.2 ± 3.5	28.2 ± 1.7	28.6 ± 5.0	38.3 ± 6.4	31.6 ± 4.5
α-Toc (μg/g)	92.1 ± 18.3	75.0 ± 8.2	41.4 ± 2.1	82.7 ± 11.4	94.9 ± 12.9	65.6 ± 7.1	48.6 ± 4.9
Female							
TBARS (μmol/g)	35.0 ± 7.1	27.9 ± 1.5	30.5 ± 3.9	33.4 ± 3.0	32.2 ± 2.7	34.1 ± 2.6	33.5 ± 2.8
α-Toc (μg/g)	97.5 ± 13.4	95.4 ± 17.7	42.8 ± 4.0	99.6 ± 16.5	67.7 ± 3.5	72.8 ± 6.6	43.2 ± 4.3

Data are represented as mean ± SEM (n = 8). SCO, scallop oil; SCO-M, SCO from Mutsu bay, Aomori, Japan; SCO-U, SCO from Uchiura bay, Hokkaido, Japan; TBARS, thiobarbituric acid reactive substances; α-Toc, α-tocopherol; TO, tuna oil.

Table S5. Growth parameters of mice administered scallop oil (SCO) for 13 weeks.

	Control			SCO-M 5%			SCO-U 5%			TO 5%		
**Male**												
Initial BW (g)	18.5	±	0.3	18.6	±	0.2	18.5	±	0.4	18.5	±	0.2
Final BW (g)	48.3	±	2.3	55.8	±	2.4	54.6	±	2.1	48.8	±	2.8
BW gain (g/day)	0.32	±	0.02	0.40	±	0.03	0.39	±	0.02	0.33	±	0.03
Food intake (g/day)	4.0	±	0.1	4.3	±	0.2	4.2	±	0.2	4.0	±	0.1
Food efficiency (g/g) [1]	0.08	±	0.01	0.09	±	0.01	0.09	±	0.01	0.08	±	0.01
Female												
Initial BW (g)	18.6	±	0.3	18.5	±	0.3	18.4	±	0.3	18.5	±	0.3
Final BW (g)	37.3	±	1.3	42.5	±	3.4	41.3	±	2.3	39.8	±	1.9
BW gain (g/day)	0.21	±	0.01	0.26	±	0.03	0.24	±	0.02	0.23	±	0.02
Food intake (g/day)	3.2	±	0.1	3.5	±	0.2	3.5	±	0.1	3.4	±	0.1
Food efficiency (g/g) [3]	0.07	±	0.01	0.07	±	0.01	0.07	±	0.01	0.07	±	0.01

Data are represented as mean ± SEM (n = 8). [1] Food efficiency (g/g) = BW gain (g/day) / food intake (g/day). BW, body weight; SCO, scallop oil; SCO-M, SCO from Mutsu bay, Aomori, Japan; SCO-U, SCO from Uchiura bay, Hokkaido, Japan; TO, tuna oil.

© 2020 by the authors. Licensee MDPI, Basel, Switzerland. This article is an open access article distributed under the terms and conditions of the Creative Commons Attribution (CC BY) license (http://creativecommons.org/licenses/by/4.0/).
